# Comparison of Enzyme Activity in Order to Describe the Metabolic Profile of Dairy Cows during Early Lactation

**DOI:** 10.3390/ijms23179771

**Published:** 2022-08-29

**Authors:** Kamila Puppel, Jan Slósarz, Grzegorz Grodkowski, Paweł Solarczyk, Piotr Kostusiak, Małgorzata Kunowska-Slósarz, Kinga Grodkowska, Anna Zalewska, Beata Kuczyńska, Marcin Gołębiewski

**Affiliations:** Institute of Animal Sciences, Departments of Animal Breeding, Warsaw University of Life Sciences, Ciszewskiego 8, 02-786 Warsaw, Poland

**Keywords:** enzymes, ketosis, acidosis, cow, AspAT, GGTP

## Abstract

Enzymatic diagnostics have practical applications in diseases of the liver, heart, pancreas, muscles, blood, and neoplastic diseases. This study aimed to compare enzyme activity to describe dairy cows’ metabolism during early lactation. Based on their general health symptoms, the cows were assigned to one of three groups: acidotic, healthy and ketotic. Samples of milk, blood and rumen fluid were collected at 12 ± 5 days postpartum. Ketotic cows were characterized by the highest malondialdehyde (MDA, 76.098 nM/mL), glutathione reductase (GluRed, 109.852 U/L), superoxide dismutase (SOD, 294.22 U/L) and gamma-glutamyltranspeptidase (GGTP, 71.175 U/L) activity. In comparing ketotic and acidotic cows, MDA, GluRed, SOD and GGTP activity were higher by a factor of almost: 1.85, 1.89, 0.79 and 2.50, respectively. Acidotic cows were characterized by the highest aspartate aminotransferase activity (AspAT, 125.914 U/L). In comparing acidotic and ketotic cows, AspAT activity was higher by a factor of almost 1.90. The use of enzymatic markers could limit the frequency of sampling for laboratory analyses and may result in a faster diagnosis of metabolic disorders. AspAT activity in blood serum seems to be a good indicator of acidosis; GGTP may participate in the pathogenesis of ketosis.

## 1. Introduction

Enzymes can bind substrates and increase their concentration locally, thus accelerating the course of the catalyzed reaction [1]. Enzymes, their substrates, and coenzymes usually do not occur uniformly within the cell but in specific spaces called compartments [2]. The compartmentation of the cell allows these compartments to be mutually isolated from each other so that opposing biochemical processes can occur within them, for example, the synthesis and the degradation of the same group of compounds [3]. The degree to which enzyme activities increase and the duration of their persistence in serum depends on the severity of pathological processes, the extent of tissue damage, and the rate of catabolism and elimination of enzymes from the plasma. The distribution of exemplary enzymes follows cytoplasm (aldolase, phosphohexose isomerase, lactate dehydrogenase, alanine aminotransferase, sorbitol dehydrogenase), mitochondria (Krebs cycle enzymes, oxidases, glutamate dehydrogenase, aspartate aminotransferase), endoplasmic reticulum (esterases, reductases, acetylases, GGTP), ribosomes (protein synthesis enzymes, ceruloplasmin, cholinesterase), and lysosomes (proteases, phosphatases, collagenases) [4,5,6,7,8].

Free radicals (oxidants) arise as a result of the actions of various factors on the matter: in the reactions of the homolytic breakdown of bonds, removal of an electron, addition or oxidation [9]. There are three stages in the reactions taking place with their participation: initiation, sonification, redox reaction, prolongation, carriers, and termination [10,11,12,13,14]. Malondialdehyde (MDA), lipid peroxidation’s end product, is considered to be a common and reliable indicator of oxidative stress [15,16]. Cells have two defense mechanisms against oxidative stress: enzymatic and non-enzymatic. Oxidoreductases are enzymes that catalyze redox reactions. There are four classes: oxidases, dehydrogenases, peroxidases, and oxygenases; for example, superoxide dismutase (SOD), glutathione peroxidase (GPx), and glutathione reductase (GluRed) [17]. GPx breaks down peroxides, protecting the body from the effects of oxidative stress. In turn, SOD participates in the reaction of disproportionation [15]. The second type of mechanism is non-enzymatic, which involves proteins with defense functions, for example, albumin and bilirubin [18]. Sordillo and Aitken [19] reported that during the physiological changes taking place in the cow’s organism, the antioxidant potential decreases, which may cause metabolic disorders resulting in oxidative stress [15,20,21]. Unfortunately, most indicator enzymes are tissue-nonspecific. Therefore, the location of lesions is based on the search for an enzyme whose level exceeds the norm by the greatest amount. Indicator enzymes include the following: aspartate aminotransferase [22], alanine aminotransferase (ALT) [23], lactate dehydrogenase [24], and creatine kinase (CK) [25]. 

Metabolic acidosis and ketosis are the primary economic issues for dairy farming due to their non-specific symptoms, difficulty obtaining a diagnosis, and reduced milk production. In cows, a rumen fluid pH of less than 5.5 for >3 h/day is consistent with a diagnosis of rumen acidosis. Ruminal fluid can be collected through a cannula that is surgically placed in the rumen [26]. Ketosis can be diagnosed by analyzing blood, urine, or milk samples. Duffield [27] reported that BHBA is a better indicator of energy imbalance in postpartum animals than NEFA, but NEFA is more useful for prepartum. Additionally, Puppel et al. [28] reported that analysis herd blood testing for β-hydroxybutyric acid (BHBA) concentration is a good method for early diagnosis of ketosis; however, it is neither cost-effective nor convenient analysis. However, previous studies have not considered the relationship between the occurrence of ketosis and acidosis and enzymatic activity.

Therefore, this study aimed to compare enzyme activity to describe the metabolism of dairy cows during early lactation.

## 2. Results

The characteristics of the acidotic, healthy, and ketotic cows are presented in Table 1. The highest concentration of milk fat characterized ketotic cows and BHBA compared to the acidotic and healthy cows. In comparing ketotic and acidotic cows, rumen fluid pH was lower by almost 0.2 fold, and reduced dry matter intake was also shown. Dry matter intake, rumen fluid pH and milk components were influenced (*p* ≤ 0.01) by the health status of cows.

**Figure 1:**
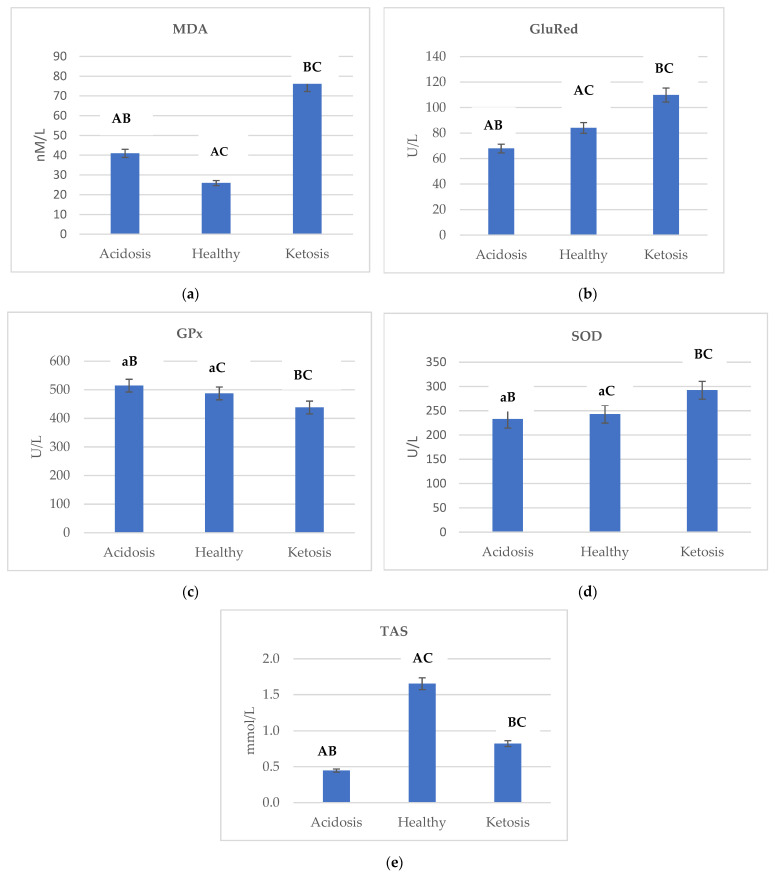
Differences in the activity of (**a**) MDA, (**b**) GluRed, (**c**) GPx, (**d**) SOD and (**e**) TAS depending on the health status of cows. Data were presented as least squares means with a standard error of the mean. Means marked with the same letters differ significantly at lowercase letters, *p* ≤ 0.05; uppercase letters, *p* ≤ 0.01. MDA, malondialdehyde; GluRed, glutathione reductase; GPx, glutathione peroxidase; SOD, superoxide dismutase; TAS, total antioxidant status; BHBA, β-hydroxybutyric acid.

Figure 1 shows the changes in the activity of (a) MDA, (b) GluRed, (c) GPX, (d) SOD, and (e) TAS depending on the health status of cows.

Ketotic cows were characterized by the highest activity of MDA, GluRed, and SOD. The lowest level of MDA in healthy cows was reported as 25.925 nM/mL, while the highest level in ketotic cows was 76.098 nM/mL–an almost three-fold higher value (Figure 1a). In comparing ketotic and acidotic cows, GluRed and SOD activity was higher by almost 1.89 and 0.79 fold, respectively (Figure 1b,d). 

In acidotic cows in relation to ketotic, GPx activity was higher by a factor of almost 1.17 (Figure 1c). Therefore, it can be concluded that GPx was affected (*p* ≤ 0.01) by the health status of cows.

Pearson correlation analysis showed a significant correlation between BHBA and oxidative stress markers (Table 2). A significant correlation was found between BHBA × MDA, BHBA × GluRed, and BHBA × SOD. A significant negative correlation was found between BHBA × GP_X_.

**Figure 2:**
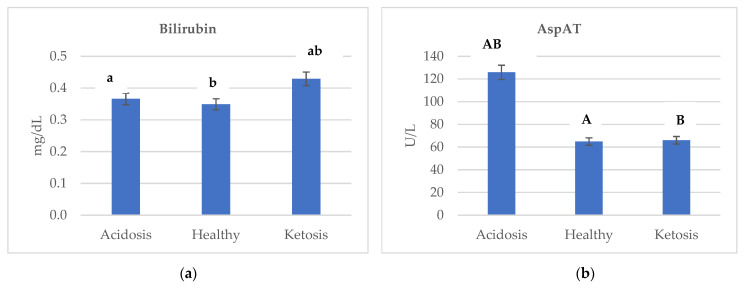

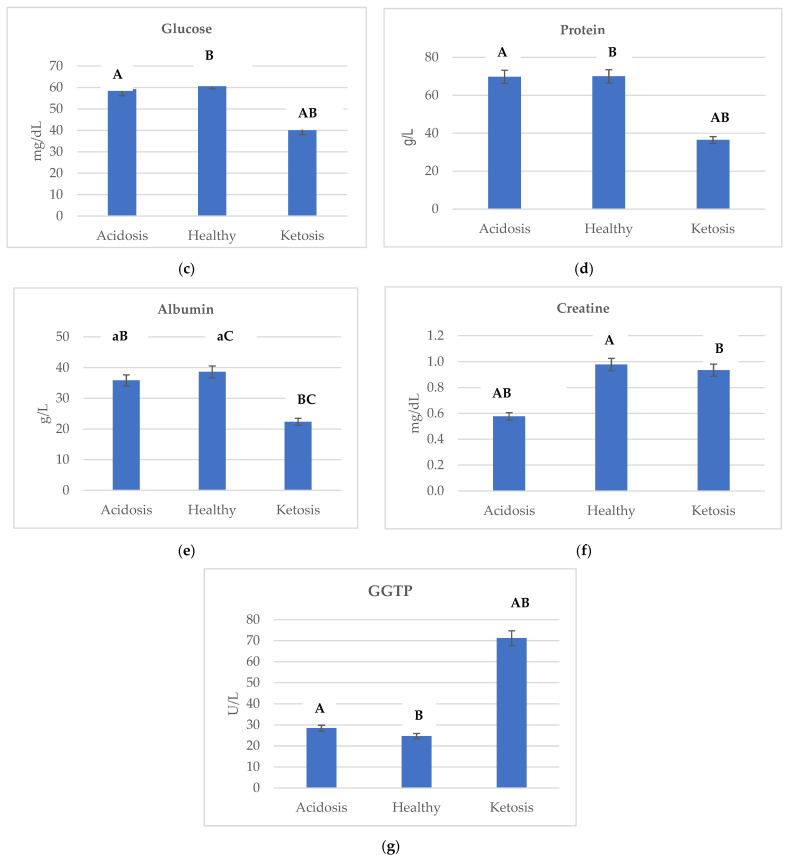
Changes in the concentration of (**a**) bilirubin, (**b**) AspAT, (**c**) glucose, (**d**) protein, (**e**) albumin, (**f**) creatine, and (**g**) GGTP depending on the health status of cows. Data were presented as least squares means with a standard error of the mean. Means marked with the same letters differ significantly at lowercase letters, *p* ≤ 0.05; uppercase letters, *p* ≤ 0.01. AspAT, aspartate aminotransferase; GGTP, gamma-glutamyltranspeptidase.

Figure 2 shows the changes in concentration of (a) bilirubin, (b) AspAT, (c) glucose, (d) protein, (e) albumin, (f) creatine, and (g) GGTP depending on the health status of cows. Ketotic cows were characterized by the highest level of bilirubin and GGTP. The lowest GGTP activity in healthy cows was reported as 24.686 U/L, while the highest in ketotic was 71.175 U/L (Figure 2g). Additionally, in comparing ketotic and acidotic cows, the concentration of bilirubin was higher by a factor of almost 1.17 (Figure 2a). 

Acidotic cows were characterized by the highest AspAT activity (125.914 U/L) compared to the ketotic and healthy cows. In comparing acidotic and ketotic cows, AspAT activity was higher by a factor of almost 1.90 (Figure 2b). AspAT was affected (*p* ≤ 0.01) by the health status of cows.

Pearson correlation analysis showed a significant correlation between BHBA and the parameters of metabolic profiles (Table 3). A significant correlation was found between BHBA × MDA, BHBA × Creatine, and BHBA × GGTP. A significant negative correlation was found between BHBA × AspAT, BHBA × Glucose, BHBA × Protein, and BHBA × Albumin.

## 3. Discussion

Ruminal acidosis has been defined when rumen pH is between 5.0 and 5.8 [29]. As shown in Table 1, the pH value of rumen fluid (*p* ≤ 0.01) was significantly lower in cows with acidosis than in healthy cows. The pH of the rumen fluid is important for developing the flora responsible for the chemical changes [30]. 

Reduced dry matter intake is also supposed to be a reliable clinical sign due to acidosis [31]. The depression in feed intake may have been caused by elevated production of unstable unsaturated fats, particularly propionate, and changes in the osmolarity in the rumen [32]. 

Oetzel [33] reported that diarrhea has been related to acidosis in dairy herds. This relationship was also confirmed by the results obtained in the present study. The increased acidity prompts sloughing of the epithelial cells in the digestive organ discharged with faces. As the hindgut gets seriously acidic, more water from epithelial cells comes to neutralize it. The result is poor consistency of the ingesta [34].

The fat content of the milk was 2.71% in cows with BHBA ranging between 0.200–0.500 mmol/L (Table 1). Nicpon and Hejlasz [35] reported that the fat percentage dropped after the induction of rumen acidosis. There are three mechanisms responsible for reducing the synthesis of fat in cases of acidosis: an increase in blood glucose, a decrease in tissue lipolysis, and reduced conversion of propionate to succinyl-CoA with a subsequent increase in the concentration of the metabolite methylmalonate (MMA) in the blood (MMA inhibits fat synthesis in mammary tissue) [36]. Thus, the fat percentage in milk seems to be a good indicator of the fermentation conditions in the rumen and for acidosis diagnosis. 

Acidosis promotes lipid peroxidation [37,38] and is involved in Reactive oxygen species (ROS) -induced intestinal inflammatory diseases [39]. Acidotic cows were characterized by the highest GPx activity (514.392 U/L) compared to ketotic and healthy cows. Mavrommatis et al. [40] found that a decrease in plasma BHBA led to increased glutathione peroxidase activity in lactating ewes. The high GPx activity appears to be a mechanism that prevents excessive peroxidation of nonesterified fatty acids transported in plasma, along with albumins, to the mammary glands [41], which is confirmed by the obtained results. In comparing acidotic and ketotic cows, the albumin concentration was almost 1.60-fold (Figure 2e). Aviram et al. [42] reported that albumin concentration is inversely related to oxidative stress, partly because they protect low-density lipoprotein and high-density lipoprotein against lipid peroxidation, along with protein carbonyl and lactoperoxidase.

AspAT is a mitochondrial enzyme. This is important in terms of diagnostics because, with slight cell damage, the serum is mainly penetrated by cytoplasmic enzymes. At the same time, during progressive destruction, they are also joined by mitochondrial enzymes [43,44]. AspAT concentrations are elevated after bruising, trauma, necrosis, infection, or neoplasia of the liver or muscle [22]. A spectrum of the hepatic disorder can also occur during heart failure; any cause of right ventricular dysfunction can be associated with severe hepatic congestion [45]. Cattle AspAT’s reference values range from 58 to 100 U/L [46]. Acidotic cows were characterized by the highest AspAT activity (125.914 U/L) compared to ketotic and healthy cows (Figure 2b). Lechowski [47] reported that bovine chronic metabolic acidosis strains the liver function, confirmed by the obtained results. Thus, AspAT activity in blood serum seems to be a good indicator of an acidosis diagnosis. 

Gamma-glutamyltranspeptidase is one of the enzymes used in diagnosing liver and biliary diseases [48] and is present on the outer surface of the plasma membrane and in blood [49]. The reference values for GGTP in cattle are in the range of 22–64 U/L [46]. Ketotic cows were characterized by the highest GGTP activity (71.175 U/L) when compared to acidotic and healthy cows (Figure 2g). Du et al. [23] reported higher serum levels for the hepatic damage markers ALT and GGTP in ketotic cows than in control cows, which is confirmed by the obtained results. GGTP is responsible for the extracellular catabolism of glutathione [50] and the catalysis of the low-density lipoprotein (LDL) oxidation [51]. During lipid peroxidation, cholesterol (to 7-oxocholesterol) and apolipoprotein B (which is the protein component of LDL particles) are oxidized [52]. The peroxidized lipids decompose, generating both free and core aldehydes and ketones [53], so GGTP may participate in the pathogenesis of ketosis. 

Ketotic cows displayed hepatic fat accumulation [23]. It should be noted that lipid accumulation impairs mitochondrial function, energy metabolism, and cellular signal transduction and in consequence reactive oxygen species overproduction [41,54]. NEFA contributes to the generation of ROS, resulting in an imbalance of oxidative species, activation of p53 transcriptional activity, inhibition of Nrf2 transcriptional activity, loss of mitochondrial membrane potential and leading to hepatocytes apoptosis [55]. Ketotic cows were characterized by the highest level of MDA (Figure 1a), GluRed (Figure 1b), and SOD (Figure 1d) activity when compared with acidotic and healthy cows, indicating that the ketotic cows displayed severe oxidative stress. Additionally, insufficient amounts of antioxidants and increased ROS decreased GPx activity [56], which is confirmed by the obtained results. 

## 4. Materials and Methods

### 4.1. Animals and Sampling

The study was conducted at the Warsaw University of Life Sciences (WULS) experimental dairy farm on a herd of approximately 370 cows maintained in a free-stall housing system. During the health monitoring procedure of all herds, 136 cows were selected and assigned to one of three groups: acidotic, healthy, or ketotic, based on their symptoms and blood serum BHBA concentrations at 12 ± 5 days postpartum, and kept separately. This made it possible to precisely analyze the dry matter intake (DMI) and the manure. DMI was determined by weighing the remaining orts. Manure was scored on a 1 to 5 basis, with a score of 1 being very fluid and 5 being extremely dry and segmented (a score of ≤2 indicated the presence of diarrhea). Body condition score (BCS) was assessed by the BCS-5 method described by Edmonson et al. [57]. Symptoms of acidosis were as follows: reduced feed intake, poor body condition score, unexplained diarrhea, lethargy, pH value of rumen fluid ≤ 5.4, BHBA 0.200–0.500 mmol/L. Ketosis: reduced feed intake, poor body condition score, pH value of rumen fluid ≥ 6.7, BHBA > 1.2 mmol/L. The characteristics of the acidotic, healthy, and ketotic cows are presented in Table 1.

The diets were balanced according to the INRA system’s recommendations. The cows’ feeding regime was based on the total mixed ration (TMR) diet (ad libitum). The ingredient composition of the TMR (kg/d DM) was as follows: maize silage, 11.05; alfalfa silage, 3.50; corn silage, 2.50; soybean meal, 2.50; pasture ground chalk, 0.20; salt, 0.05; rapeseed meal, 2.10; and magnesium oxide, 0.06. The remaining factors characterizing TMR were as follows: total kg of DM, 21.20; daily intake (kg), 19.90; Netto energy lactation (Mcal/kg), 1.75; average milk production (kg), 37.02; a unit of milk production balance (%), 3.45; protein digested in the small intestine when rumen-fermentable nitrogen is limiting, 2.51; and protein digested in the small intestine when rumen-fermentable energy is limiting, 2.23. Cows were fed twice a day.

Samples of milk, blood and rumen fluid were collected at 12 ± 5 days postpartum, resulting in 136 samples of both milk, blood and rumen fluid. Milk yield was recorded daily and individual milk samples were taken for milk composition analyses. The milk samples (250 mL) were obtained from each cow using milk samplers (from the morning and evening milking), placed in sterile bottles, and transported to the Milk Testing Laboratory of WULS for compositional analysis. 

The blood samples (10 mL) were obtained via jugular vein puncture using a tube (Vacuette, Essen, Germany), then centrifuged at 1800× *g* at 4 °C for 15 min, and the supernatant was immediately transported to the Veterinary Centre of WULS.

Rumen fluid samples were collected at 6 h after the morning feeding at 12 ± 5 days postpartum using the oral stomach tubing (OST) technique by a veterinarian. The OST sampling device consisted of a 250 cm long orogastric tubing with a 15 mL perforated plastic conical tube attached to one end. On the other end, the OST has been connected to the vacuum pump. Approximately 200 mL of initially sampled ruminal fluid was discarded, and an additional 200 mL of ruminal fluid was collected and processed for further analysis.

### 4.2. Chemical Analyses

The basic parameters of milk, i.e., fat and protein, were determined via automated infrared analysis using a Milkoscan FT 120 analyzer (Foss Electric, Hillerød, Denmark). 

The MDA levels in blood plasma were determined using a NanoQuant Infinite M200 Pro analyzer (Tecan Austria GmbH, Grödig, Austria) at a wavelength of 532 nm according to the methodology described by Kapusta et al. [16]. To 250 µL of blood plasma, 25 µL of 0.2% 2,6-bis (1,1-dimetyoetylo)-4-metylofenol (Sigma-Aldrich, Warsaw, Poland) and 1 mL of 5% trichloroethanoic acid (Sigma-Aldrich, Warsaw, Poland) was added. After centrifugation (14,000× *g* for 10 min), 750 µL of clear supernatant was transferred to a glass tube, and 500 µL of 0.6% thiobarbituric acid (Sigma-Aldrich, Warsaw, Poland) was added, then mixed and incubated for 45 min in a water bath in 90 °C. After cooling on ice and centrifuging at 4000× *g* for 5 min, 200 µL of clear supernatant was transferred to the microplate. 

The amount of GluRed, GPx, SOD, and TAS (total antioxidant status) in blood plasma was determined using a NanoQuant Infinietie M200Pro analyzer (Tecan Austria GmbH, Grödig, Austria) using a dedicated ELISA Kit, according to the methodology described by RANDOX (Randox Laboratories, Crumlin, UK), respectively: Glutathione Reductase Cat no GR2608, Ransel (glutathione peroxidase) Cat no SC692, Ransod (superoxide dismutase) Cat no SD126, Total Antioxidant Status Cat no NX2331. 

Total antioxidant status, the incubation of ABTS^®^ (2,2′-Azyno-di-[sulfonian 3-etylbenztiazoliny]) with peroxidase (metmyoglobin) leads to the formation of the radical cation ABTS ++. This substance is blue-green and can be detected at a wavelength of 600 nm. Antioxidants present in the sample reduce the development of the blue-green color in proportion to their concentration (Randox Laboratories, Crumlin, UK).

HX − Fe^III^ + H_2_O_2_ → X − [Fe^IV^ = 0] + H_2_O

ABTS^®^ + X − [Fe^IV^ = 0] → ABTS^®+^ + HX − Fe^III^

Superoxide dismutase was measured by the degree to which the xanthine oxidase and superoxide radicals inhibited the reaction (Randox Laboratories, Crumlin, UK):

O_2_^•^ + O_2_^•^ + 2H^+SOD^ → O_2_ + H_2_O_2_

Glutathione peroxidase catalyzes the oxidation of glutathione by cumene hydroperoxide, while Glutathione reductase catalyzes the reduction of glutathione (Randox Laboratories, Crumlin, UK):

2GSH + ROOH ^GPx^ → ROH + GSSG + H_2_O

GSSG + NADPH + H^+GR^ → NADP^+^ + 2GSH

The level of Bilirubin, BHBA, AspAT, Glucose, Protein, Albumin, Creatine, and GGTP was determined using a BS800M biochemical analyzer (PZ Cormay, Warsaw, Poland) in the Veterinary Centre of WULS. 

Samples of the ruminal fluid were analyzed for pH in the Veterinary Centre of WULS.

### 4.3. Statistical Analysis

The data were compiled statistically via an analysis of variance (ANOVA) using the least-squares method and PS IMAGO PRO 7.0 [58]. Significant differences among group means were calculated using the F statistic. The distribution of both metabolic and oxidative stress parameters was examined using the Shapiro–Wilk test. The ANOVA analysis was used to establish the influence of BHBA levels on metabolic and oxidative stress parameters. Pearson’s correlation coefficients were also calculated to find the relationship between BHBA and metabolic and oxidative stress markers.

## 5. Conclusions

The use of enzymatic markers could limit the frequency of sampling for laboratory analyses and may result in faster diagnosis of metabolic disorders. The degree to which enzyme activities increase depends on the health status of cows. Based on these results, it can be concluded that ketotic cows displayed severe oxidative stress. AspAT activity in blood serum seems to be a good indicator of acidosis. On the other hand, GGTP may participate in the pathogenesis of ketosis.

## Figures and Tables

**Table 1:** Characteristics of the acidotic, healthy, and ketotic cows.

	Acidosis (n = 46)		Healthy (n = 42)		Ketosis (n = 48)		*p*-Value
	LSM	SEM	LSM	SEM	LSM	SEM	
Milk Parameters							
Milk Yield [kg/d]	26.664	1.020	29.147	1.341	31.083	0.897	*p* ≤ 0.01
Protein [%]	3.608	0.043	3.640	0.056	3.124	0.038	*p* ≤ 0.05
Fat [%]	2.71	0.124	4.27	0.163	5.12	0.109	*p* ≤ 0.01
BHBA [mmol/L]	0.200–0.500	0.019	0.51–1.2	0.025	>1.2	0.017	*p* ≤ 0.01
pH value of rumen fluid	≤5.4	0.102	6.6	0.121	6.7	0.109	*p* ≤ 0.01
DMI	17.25	0.143	20.01	0.174	15.87	0.111	*p* ≤ 0.01
BCS	2.10	0.026	3.30	0.064	2.34	0.052	*p* ≤ 0.01
Diarrhea	+		−		−		
Parity	2nd and 3rd		2nd and 3rd		2nd and 3rd		-

BHBA, β-hydroxybutyric acid; DMI, Dry matter intake; LSM, Least square mean; BCS, Body Condition Score; SEM, Standard error of LSM and *p*-value, Probability value.

**Table 2:** Pearson correlations between BHBA and markers of oxidative stress.

	MDA	GluRed	GPx	SOD	TAS	BHBA
MDA	1	0.308 **	0.013	0.167 *	−0.281 **	0.587 **
GluRed		1	0.066	0.102	0.102	0.495 **
GP_X_			1	−0.034	−0.120	−0.164 *
SOD				1	−0.036	0.220 **
TAS					1	−0.041
BHBA						1

** Correlation significant at a level of 0.01 (two-sided). * Correlation significant at a level of 0.05 (two-sided). MDA: malondialdehyde; GluRed: glutathione reductase; GPx: glutathione peroxidase; SOD: superoxide dismutase; TAS: total antioxidant status; BHBA: β-hydroxybutyric acid.

**Table 3:** Pearson correlations between BHBA and the parameters of metabolic profiles.

	MDA	BHBA	Bilirubin	AspAT	Glucose	Protein	Albumin	Creatine	GGTP
MDA	1	0.587 **	0.188 **	−0.049	−0.689 **	−0.656 **	−0.604 **	−0.008	0.758 **
BHBA		1	0.113	−0.362 **	−0.660 **	−0.687 **	−0.680 **	0.324 **	0.747 **
Bilirubin			1	−0.114	−0.212 **	−0.154 *	−0.007	−0.001	0.201 **
AspAT				1	0.129	0.178 *	0.093	−0.449 **	−0.191 **
Glucose					1	0.688 **	0.668 **	−0.046	−0.790 **
Protein						1	0.796 **	−0.099	−0.713 **
Albumin							1	0.036	−0.742 **
Creatine								1	0.093
GGTP									1

** Correlation significant at a level of 0.01 (two-sided). * Correlation significant at a level of 0.05 (two-sided). MDA, malondialdehyde; BHBA, β-hydroxybutyric acid; AspAT, aspartate aminotransferase; GGTP, gamma-glutamyltranspeptidase.

## Data Availability

All data generated or analyzed during the study are included within the article.
